# *THBD* sequence variants potentially related to recurrent pregnancy loss

**DOI:** 10.1186/s12958-017-0311-0

**Published:** 2017-12-01

**Authors:** Paula Quintero-Ronderos, Eric Mercier, Jean-Christophe Gris, Clara Esteban-Perez, Harold Moreno-Ortiz, Dora Janeth Fonseca, Elkin Lucena, Daniel Vaiman, Paul Laissue

**Affiliations:** 1 Center For Research in Genetics and Genomics-CIGGUR, GENIUROS Research Group, School of Medicine and Health Sciences, Universidad del Rosario, Carrera 24 N° 63C, -69 Bogotá, Colombia; 2 Department of Haematology, University Hospital, Nîmes, France; 3 Faculty of Pharmacy and Biological Sciences and Research Team EA 2992, University of Montpellier, Montpellier, France; 4 Department of Reproductive Genetics, Fertility and Sterility Colombian Center, Bogotá, Colombia; 5 Institut Cochin, Université Paris Descartes, CNRS (UMR 8104), Paris, France; 6 Inserm, U1016, Paris, France

**Keywords:** Recurrent pregnancy loss, THBD, Molecular marker, Female infertility

## Abstract

Recurrent pregnancy loss (RPL) is a frequently occurring disease, which is classified as idiopathic in more than 50% of cases. THBD, the endothelial cell receptor for thrombin, has been associated with distinct biological processes and considered a coherent RPL-related candidate gene. In the present study, we have sequenced the complete coding region of *THBD* in 262 patients affected by RPL. Bioinformatics analysis and screening of controls strongly suggested that the THBD-p.Trp153Gly mutation might be related to RPL aetiology. It could be used, after its validation by functional assays, as a molecular marker for diagnostic/prognostic purposes.

## Introduction

Recurrent pregnancy loss (RPL), which is clinically defined as at least two consecutive pregnancy losses prior to 20 weeks of gestation, is a frequently occurring disease. Although several RPL’s aetiologies have been described, the disease is still classified as idiopathic in more than 50% of cases, which underlines the potential involvement of genetic factors. However, only a few studies have revealed links between specific genetic variants and RPL’s origin.

THBD, the endothelial cell receptor for thrombin, has been associated with distinct biological processes and considered a coherent RPL-related candidate gene [1]. It has been largely studied regarding its involvement in the natural anticoagulation system. Its binding to thrombin leads to decreased fibrinogen-to-fibrin conversion and activation of the coagulation cascade (e.g. factors V, VIII, platelets). The THBD/thrombin complex catalyses protein C activation which, in turn, increases inhibition of the coagulation effect via cofactor Va and VIIIa inactivation. These features have been well documented in vivo as *Thbd* knock-out mice have displayed embryonic death secondary to coagulation dysfunction at the foetal-maternal interface [2]. A THBD truncating mutation in humans (p.Cys537X), which disturbs transmembrane domain conformation and has eliminated the cytoplasmic domain, has been functionally related to an autosomal dominant bleeding disorder [3]. Regarding human pregnancy dysfunction, it has been postulated that RPL might be associated with relative resistance to THBD as well as to its decrease in placental tissue. However, studying a potential association between some *THBD* SNP and RPL has led to contradictory results.

In the present study, we have sequenced the complete coding region of *THBD* in 262 patients affected by RPL. Bioinformatics analysis and screening of controls strongly suggested that the THBD-p.Trp153Gly mutation might be related to RPL aetiology. It could be used, after their validation by functional assays, as a molecular marker for diagnostic/prognostic purposes.

## Methods

### Patients and controls

Two hundred and sixty two women affected by RPL were included in the study. 233 and 29 were of French (Caucasian) and Colombian (Mestizo) origin, respectively. French women affected by RPL formed part of a previously-established group (the Nîmes Obstetricians and Haematologists patient cohort) of women suffering pregnancy loss [4]. Colombian women suffering RPL attended the Center For Research in Genetics and Genomics (Universidad del Rosario, Bogotá, Colombia). They presented normal karyotypes, and were affected by at least 2 pregnancy losses. They did not display antecedents of coagulation, autoimmune (e.g. antiphospholipid syndrome) and/or metabolic disorders. Autoimmune and coagulation disorders were excluded by biochemical tests. Uterine morphology was normal. All cases lack familial antecedents of consanguinity or RPL. No extensive genotyping initiatives at genome scale have been carried out in Colombian population. Thus, to screen interesting variants identified in RPL and Colombian patients, we have recruited 165 Colombian (Mestizo) women under 50 years old having at least one child and lacking antecedents of medical complications during pregnancy. We did not include Caucasian control women for *THBD* sequencing because public databases of SNPs are enriched by data of individuals from this ethnical origin. All the experimental steps of this study were approved by the Universidad del Rosario’s Ethics Committee, and was conducted in line with the Declaration of Helsinki (approval date: 1 February 2017. Institional Review Board reference number: CEI-DVN021–1-063).

### THBD molecular and bioinformatics analysis

We extracted genomic DNA from whole blood samples by using the standard salting-out procedure. Then, DNA was used to amplify (PCR) the complete *THBD* open reading frame. Amplicons were purified by using shrimp alkaline phosphatase and exonuclease I. Sequencing involved using internal primers. Sequences were compared to the *THBD* wild type version (ENSG00000178726). Primer sequences, PCR and sequencing technical conditions are available upon request.

The frequency of *THBD* sequence variants in distinct healthy populations was screened using the Ensembl database. Allele frequencies of the c.1418 C > T (p.Ala473Val) variant in European controls were obtained from the Ensembl database and compared to that found in our French RPL women. Allele frequencies of variants identified in RPL Colombian patients were compared to those from a control group (*n* = 165) from the same ethnical origin. SIFT and PolyPhen bioinformatics tools were used for predicting potential deleterious effects of missense variants. Protein alignments between species were performed to assess the conservation during evolution of interchanged residues.

### Statistical analysis

We performed chi-square tests for calculating significant differences of allele frequencies between cases and controls individuals.

## Results

RPL patients were divided in different subgroups (G1 through G4) according to the number of miscarriages: G1 (2 RPLs), G2 (3 RPLs), G3 (4 RPLs) and G4 (> 4 RPLs). Most RPL patients (59.9%, *n* = 157) belonged to the G1. 26.3% (*n* = 69) and 6.9% (*n* = 18) of patients were classified into the G2 and G3 groups, respectively. G4 included 18 RPL patients (6.9%). Screening for mutations of the *THBD* gene displayed 2 non-synonymous sequence variants: c.457 T > G (p.Trp153Gly) and c.1418 C > T (p.Ala473Val) (Table 1). *THBD*-c.457 T > G and *THBD*-c.1418 C > T displayed, in the SNP Ensembl database, minor allele frequencies (MAF) of 0.00 and 0.16, respectively. For the c.457 T > G variant, which was found uniquely in Colombian patients, statistically significant differences in allele frequencies were identified between cases and controls from the same ethnical origin (*p* = 9 × 10^−6^). For the c.1418 C > T variant, a statistically significant difference (*p* = 0.02) was identified between French RPL patients and European controls recorded in the Ensembl database. Conversely, no differences in allelic frequencies for the c.1418 C > T variant were identified between Colombian RPL patients and control individuals from the same ethnical origin. The c.457 T > G and c.1418 C > T variants were most frequently found in the G1, in both French and Colombian RPL women (Table 1).

**Table 1:** *THBD* open reading frame sequencing in RPL patients and control individuals

French RPL patients													
DNA	Protein	Zygocity (n)	Rs	RPL Group	Patients	European controls (Ensembl)	MAF	Allelic Frequency (cases)	Allelic Frequency (controls)	*P* value	SIFT	PolyPhen	Evolutive conservation
c.1418 C > T	p.Ala473Val	Homozygote (3)	rs1042579	G1: 65,6%	32/233	173/503	0.16	C: 0,92 T: 0,08	C: 0,80 T: 0,20	0,02	Tolerated (0,42)	Bening (0)	Yes
		Heterozygote (29)		G2: 25%									
				G3: 9,4%									
Colombian RPL patients													
DNA	Protein	Zygocity (n)	Rs	RPL Group	Patients	Colombian Controls	MAF	Allelic Frequency (cases)	Allelic Frequency (controls)	*P* value	SIFT	PolyPhen	Evolutive conservation
c. 457 T > G	p.Trp153Gly	Homozygote (3)		G1: 50%	4/29	0/165		T: 0,88 G: 0,12	T: 1 G: 0	0,000009	Deleterious (0)	Probably damaging (0.976)	Yes
		Heterozygote (1)		G3: 25%									
				G4: 25%									
c.1418 C > T	p.Ala473Val	Heterozygote (4)	rs1042579	G1: 50%	4/29	51/165	0.16	C: 0,93 T: 0,07	C: 0,95 T: 0,05	0,73	Tolerated (0,42)	Bening (0)	Yes
				G2: 25%									
				G4: 25%									

## Discussion

In the present study, we focused our attention on the research of rare *THBD* non-synonymous mutations underlying theoretically putative moderate/strong functional effects contributing to RPL. We consider that p.Trp153Gly is particularly interesting as low MAF was reported in public databases of SNP and significant statistical differences were found in allele frequencies between cases and controls of Colombian origin. The p.Trp153Gly mutation involved the exchange of an aminoacid conserved during evolution (Trp^153^), and Trp to Gly exchange evoked a drastic modification of protein’s local physicochemical properties. In addition, SIFT and Polyphen scores were compatible with deleterious effects. These features might be related to an improper folding of the protein leading to functional disturbances.

Interestingly, this mutation was located at the end of the C-type lectin-like (CLL) module, which has been associated with protective properties against inflammatory damage [5]. Due to its location at the N-terminal extracellular region of the protein, it would seem proper for the CLL domain to interact with other molecules and cells. Transgenic mice lacking the Thbd-CLL region have displayed enhanced synthesis of cytokines and a significant inflammation reaction when treated with lipopolysaccharide [5]. It has also been proposed that the THBD-CLL domain participates in cell-cell adhesion by maintaining the integrity of endothelial junctions and contributing to maintaining blood vessels’ quiescent state [6, 7]. CLL’s anti-inflammatory properties have been related to HMGB1 (high mobility group box 1) functions, such molecule originating in necrotic and inflammatory cells and displaying pro-inflammatory mediator properties [5]. The CLL domain of THBD binds directly to HMGB1, interfering with its binding to specific receptors and decreasing inflammation. We thus hypothesise that the THBD-p.Trp153Gly mutation might perturb the interaction between THBD and HMGB1, thereby leading to local enhanced inflammation at the foetal-placental interface and contributing towards miscarriage aetiology. We cannot discard a decrease of mutant THBD bioavailability at the cell surface secondary to abnormal protein folding and cytoplasmic trafficking. We consider therefore that the p.Trp153Gly mutation might be a relevant molecular marker for RPL in Colombian population. Concerning the c.1418 C > T (p.Ala473Val) variant (located in the epidermal growth-factor–like repeats region, EGF-LR), it is difficult to associate it with a potential deleterious effect since, although statistical differences were identified in allelic frequencies between cases and controls from French origin, no differences were observed in individuals from Colombian origin. A lack of association of this variant with the RPL phenotype was reported by Kaare et al., 2007 and Guerra-Shinohara et al., 2012 [8, 9]. Conversely, Cao et al., 2013 reported that this variant was significantly associated with the increased risk of RPL in Chinese population [10]. These differences might be due to a small population size in some studies and/or secondary to genetic ethnically specific differences. Thus, we cannot discard that the c.1418 C > T variant confers susceptibility to RPL. To note, this variant has been associated with thrombosis [11–15].

The mutations found in the present study might alter different mechanisms related to complement activation, coagulation, fibrinolysis, inflammation and cell/cell adhesion. Dysfunction of the CLL domain might be related to enhanced susceptibility to inflammation and alterations in the trophoblast/endometrium adhesion. Mutations in the EGF-LR domain might lead to expression disturbances of key molecules such as the activated protein C and TAFI, promoting an hypercoagulation state.

In our study the c.457 T > G and c.1418 C > T variants were most frequently identified in women affected by 2 miscarriages. However, due to the complexity of the RPL phenotype, in which numerous genes (and mutations) are probably involved, it was difficult to establish an accurate genotype-phenotype correlation. For clinicians, these variants might be used in the near future to establish more accurate treatment for women affected by RPL and genetic counselling.

## Conclusions

In synthesis our findings argues in favour of an aetiological link between THBD sequence variants and RPL aetiology. We consider that the THBD-p.Trp153Gly mutation might be related to RPL aetiology in Colombian RPL women. It could be used, after its validation by functional assays, as a molecular marker for diagnostic/prognostic purposes.

## Abbreviations

CLL: C-type lectin-like
EGF-LR: epidermal growth-factor–like repeats region.
HMGB1: high mobility group box 1
MAF: minor allelic frequency
RPL: recurrent pregnancy loss
SNP: single nucleotide polymorphism
THBD: thrombomodulin
